# Antibiotic resistance rates and physician antibiotic prescription patterns of uncomplicated urinary tract infections in southern Chinese primary care

**DOI:** 10.1371/journal.pone.0177266

**Published:** 2017-05-09

**Authors:** Carmen Ka Man Wong, Kenny Kung, Philip Lung Wai Au-Doung, Margaret Ip, Nelson Lee, Alice Fung, Samuel Yeung Shan Wong

**Affiliations:** 1 JC School of Public Health and Primary Care, Division of Family Medicine and Primary Health Care, Faculty of Medicine, The Chinese University of Hong Kong, Hong Kong, China; 2 Department of Microbiology, Faculty of Medicine, The Chinese University of Hong Kong, Hong Kong, China; 3 Department of Medicine and Therapeutics, Faculty of Medicine, The Chinese University of Hong Kong, Hong Kong, China; California Northstate University College of Medicine, UNITED STATES

## Abstract

Uncomplicated urinary tract infections (UTI) are common in primary care. Whilst primary care physicians are called to be antimicrobial stewards, there is limited primary care antibiotic resistance surveillance and physician antibiotic prescription data available in southern Chinese primary care. The study aimed to investigate the antibiotic resistance rate and antibiotic prescription patterns in female patients with uncomplicated UTI. Factors associated with antibiotic resistance and prescription was explored. A prospective cohort study was conducted in 12 primary care group clinics in Hong Kong of patients presenting with symptoms of uncomplicated UTI from January 2012 to December 2013. Patients’ characteristics such as age, comorbidity, presenting symptoms and prior antibiotic use were recorded by physicians, as well as any empirical antibiotic prescription given at presentation. Urine samples were collected to test for antibiotic resistance of uropathogens. Univariate analysis was conducted to identify factors associated with antibiotic resistance and prescription. A total of 298 patients were included in the study. *E*. *coli* was detected in 107 (76%) out of the 141 positive urine samples. Antibiotic resistance rates of *E*. *coli* isolates for ampicillin, co-trimoxazole, ciprofloxacin, amoxicillin and nitrofurantoin were 59.8%, 31.8%, 23.4%, 1.9% and 0.9% respectively. *E*. *coli* isolates were sensitive to nitrofurantoin (98.1%) followed by amoxicillin (78.5%). The overall physician antibiotic prescription rate was 82.2%. Amoxicillin (39.6%) and nitrofurantoin (28.6%) were the most common prescribed antibiotics. Meanwhile, whilst physicians in public primary care prescribed more amoxicillin (OR: 2.84, 95% CI: 1.67 to 4.85, *P*<0.001) and nitrofurantoin (OR: 2.01, 95% CI: 1.14 to 3.55, *P* = 0.015), physicians in private clinics prescribed more cefuroxime and ciprofloxacin (*P*<0.05). Matching of antibiotic prescription and antibiotic sensitivity of *E*. *coli* isolates occurred in public than private primary care prescriptions (OR: 6.72, 95% CI: 2.07 to 21.80 *P* = 0.001) and for other uropathogens (OR: 6.19, 95% CI: 1.04 to 36.78 *P* = 0.034). Mismatching differences of antibiotic prescription and resistance were not evident. In conclusion, nitrofurantoin and amoxicillin should be used as first line antibiotic treatment for uncomplicated UTI. There were significant differences in antibiotic prescription patterns between public and private primary care. Public primary care practitioners were more likely to prescribe first line antibiotic treatment which match antibiotic sensitivity of *E*. *coli* isolates and other uropathogens. Further exploration of physician prescribing behaviour and educational interventions, particularly in private primary care may helpful. Meanwhile, development and dissemination of guidelines for primary care management of uncomplicated UTI as well as continued surveillance of antibiotic resistance and physician antibiotic prescription is recommended.

## Introduction

Urinary tract infections (UTI) are one of the most common bacterial infections in primary care and classified as either complicated or uncomplicated [1]. Uncomplicated UTI occur in healthy individuals with either no structural or functional abnormalities of the urinary tract [1]. Bacterial pathogens are thought to be the cause of the presenting symptoms, and are often treated with antibiotics, which accounts for up to 95% of the antibiotic prescription for UTI in primary care settings [2]. *Escherichia coli (E*. *coli)* is the most common pathogen isolated in around 75% of uncomplicated UTI [3,4]. However, cases may be complicated by the *E*. *coli* producing extended-spectrum β-lactamase (ESBL), resulting in resistance to multiple β-lactam antibiotics [3]. Three international surveillance studies estimated that the prevalence of *E*. *coli* resistant to several types of antibiotics were around 8% (nitrofurantoin) to 48% (ampicillin) in North American, South American and European populations [4–6].

Meanwhile, primary care physicians are called to be antimicrobial stewards. Antibiotic resistance has been shown to be associated with patients’ age and sex, previous antimicrobial therapy and can be dynamic, responding to patterns of antibiotic treatment [7,8]. It is important to understand antibiotic prescribing behaviour of primary care physicians and antibiotic resistance patterns in primary care. Many studies has been conducted in the UK [8,9], however, there has been few studies to explore physician prescribing behaviour and antibiotic resistance in a Chinese primary care population. Antibiotic resistance of uropathogens and the prevalence of ESBL production of community-acquired UTI in Hong Kong is high (6.6% and 10% in 2004 and 2005 respectively) [10]. This study aimed to explore (1) the antibiotic resistance of urinary isolates of symptomatic patients presenting to primary care, (2) empirical antibiotic prescribing behaviours of primary care physicians and (3) factors associated with antibiotic resistance and physician antibiotic prescription.

## Materials and methods

### Design and setting

This was a prospective cohort study of public and private primary care clinics in Hong Kong. Public primary care clinics (government funded group primary care clinics) and private clinics provide more than 80% of primary care services for general population in Hong Kong [11]. Invitations were sent to all public and private group teaching practices in the New Territories and Kowloon region from January 2012 to December 2013. Group practices were chosen to increase yield of urinary samples. New Territories and Kowloon regions were selected to facilitate transportation of specimens.

### Characteristics of patients

Eligible patients were women above the age of 16 presenting to primary care clinics with symptoms suggestive of UTI. The attending physician assessed eligibility criteria and invited patients with a provisional diagnosis of uncomplicated UTI based on UTI symptoms to participate in the study. Patients who were already on antibiotics or had antibiotics in the preceding 21 days, pregnant, having functional or structural abnormalities of the urinary tract, immunocompromised illness (e.g. AIDS, leukaemia, active viral hepatitis, multiple myeloma) or using immunosuppressants (e.g. oral corticosteroids, medication for modification of autoimmune conditions or transplanted organ and chemotherapy) were excluded from the study. Physicians were required to fill in a case report which included patient’s demographics, symptoms of UTI, hospitalisation history, antibiotic prescription for the present UTI, records of previous bacterial pathogens in the past one year and antibiotics prescribed in the past one year. Chronic medical conditions (e.g. diabetes mellitus, hypertension, ischaemic heart disease, chronic obstructive pulmonary disease and asthma, thyroid disease) were also recorded.

### Ethical statement

This study was approved by the Clinical Research Ethics Committee of the Chinese University of Hong Kong and New Territories East Cluster of the Hospital Authority [CRE-2011.115]. Written formal consent was obtained from all participants.

### Laboratory analysis

Participants were given verbal instructions on the steps of collecting a clean catch mid-stream urine (MSU) sample. The sample was collected in a sterile container refrigerated and delivered within the same day to the centralized accredited University Pathology laboratory for processing. Only one sample per patient was obtained.

In order to identify the major pathogen of UTI, the collected MSU specimens were processed for urine microscopy and cultures. Urine microscopy was performed by standard methods according to laboratory operating procedures (PHLS BSOP41, 1998; Urine for Microscopy, bacterial counts, identification and antibiotic susceptibilities, RCPA QAP Ltd). The urine sample was mixed well and a known quantity was examined under the inverted microscope at 20X power field in a flat-bottomed microtitre tray. The number of red and white cells were enumerated and reported as negative, moderate (10,000–100,000 cells/ml), large number (>100,000 cells/ml) of cells. Specimens were inoculated by the paper strip method (MAST) onto chromogenic medium (CPSE plate, Biomerieux) and incubated aerobically for 18–24 h at 35°C. The number of colonies were enumerated and reported with 0, <10^3^, 10^3−4^, 10^4−5^, and >10^5^ cfu/ml as no growth, insignificant, scanty, moderate and heavy growth respectively. Only urine cultures with heavy growth of a single bacterial type (>10^5^ cfu/ml) were included in the analysis.

To identify antibiotic resistance patterns in the urinary isolates, the samples were further tested for antibiotic susceptibility to amoxicillin, ampicillin, ciprofloxacin, co-trimoxazole, gentamicin and nitrofurantoin and to detect ESBL positivity. Antibiotic susceptibilities were performed and interpreted as according to Clinical and Laboratory Standards Institute (CLSI) (Clinical and Laboratory Standards Institute. Performance Standards for Antimicrobial Susceptibility Testing. CLSI document. Wayne). Specimens which were not processed immediately were stored at -4°C.

### Data analysis

Descriptive statistics (mean±SD, percentages) were used to describe the frequency of bacterial pathogens, antibiotic resistance and prescription pattern.

Chi-squared test and univariate analysis were used for statistical analysis. *E*. *coli* antibiotic resistance was stratified with age group and chronic medical conditions and analysed by chi-square test. Odds ratios (OR) with 95% confidence intervals (95% CI) were calculated to identify factors associated with *E*. *coli* isolates resistance to antibiotics and for antibiotic prescription. A two tailed *P*< 0.05 was considered significant and all statistical analyses were performed by using SPSS v 21.0 Window (IBM Inc.,SPSS Inc, Chicago, IL USA).

## Results

### Patients and urine samples characteristics

Of the invited group clinics, 5 public and 12 private group clinics participated in the study and 306 female patient presentations were recruited into the study. Of these, eight clinical presentations were from previously recruited patients and only their first presentation were included in the study. Thus a total of 298 patients participated in the study of 145 (48.7%) and 153 (51.3%) were recruited from public and private clinics respectively (Table 1). The mean age of patients was 53.8±17.1 years old.

**Table 1:** Patients’ characteristics (n = 298).

Patients’ characteristics		n (%)/mean±SD^a^		
		Total (n = 298)	Public (n = 145)	Private (n = 153)
**Age**	All	53.8±17.1	58.3±15.3	49.5±17.7
	17–35	45 (15.1%)	11 (7.6%)	34 (22.2%)
	36–50	75 (25.2%)	29 (20%)	46 (30.1%)
	51–64	100 (33.6%)	57 (39.3%)	43 (28.1%)
	≥65	78 (26.2%)	48 (33.1%)	30 (19.6%)
**Occupation**	Professionals/ Officers/ Technicians	16 (5.4%)	7 (4.8%)	9 (5.9%)
	Server/Clericals	62 (20.8%)	28 (19.3%)	34 (22.2%)
	Housewife/Unemployed	107 (35.9%)	69 (47.6%)	38 (24.8%)
	Retired	49 (16.4%)	31 (21.4%)	18 (11.8%)
	Students	13 (4.4%)	3 (2.1%)	10 (6.5%)
	Missing	51 (17.1%)	7 (4.8%)	44 (28.8%)
**Chronic medical conditions**	Hypertension	83 (27.9%)	54 (37.2%)	29 (19.0%)
	Diabetes	49(16.4%)	33(22.8%)	16(10.5%)
	Asthma	4 (1.3%)	3 (2.1%)	1 (0.7%)
	Ischemic heart disease	9 (3%)	3 (2.1%)	6 (3.9%)
	Thyroid disease	6 (2%)	2 (1.4%)	4 (2.6%)
	COPD	1 (0.3%)	1 (0.7%)	0
	Gout	1 (0.3%)	1 (0.7%)	0
**Number of chronic medical conditions**	0	189 (63.4%)	73 (50.3%)	116 (75.8%)
	1	48 (16.1%)	32 (22.1%)	16 (10.5%)
	≥2	61 (20.5%)	40 (27.6%)	21 (13.7%)

^a^Data were presented as mean±SD for continuous variables and n (%) for categorical variables.

**Fig 1:**
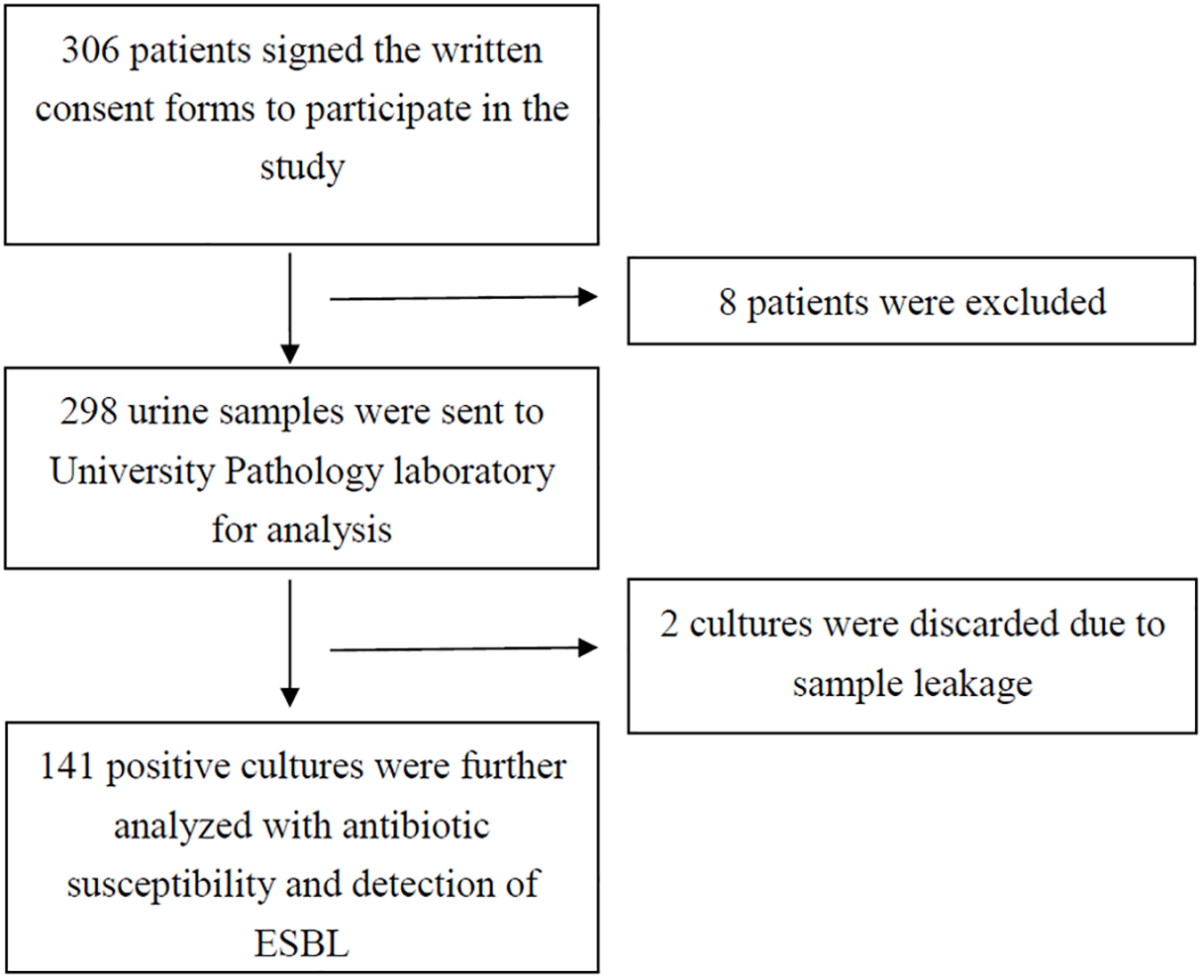
Flow chart of the patient’s recruitment process and analysis of their urine samples.

Of the samples received, two were discarded as there was leakage on transit and could not be processed. A positive urine culture was found in 141 (47.3%) of the collected samples (Fig 1) whilst 73 (24.5%) showed no growth, 68 (22.8%) as insignificant growth and 14 (4.7%) showed mixed bacteria and probable contamination.

### Bacterial pathogens among positive urine isolates

Among the 141 positive urine isolates, the most common uncomplicated UTI pathogen was *E*. *coli* (75.9%). Other uropathogens included *Klebsiella* (9.2%), *Enterococcus* (3.5%), *Proteus Mirabilis* (3.5%) and *Citrobacter* (2.8%).

*E*. *coli* infection occurred mostly in women who aged 51 above (63.5%) and most likely to present with two or more symptoms (79.5%) of which dysuria, frequency and urgency were common symptoms experienced (Table 2).

**Table 2:** Patients’ characteristics of those with uncomplicated UTI with *E*. *coli* infection (n = 107).

Patients’ characteristics		n (%)
**Age**	**mean±SD**^**a**^ 56.3±17.9	
	17–35	13 (12.1%)
	36–50	26 (24.3%)
	51–64	38 (33.6%)
	65 and above-80	34 (31.8%)
**Symptoms**	Dysuria	89 (83.2%)
	Frequency	71 (66.4%)
	Urgency	48 (44.9%)
	Abdominal pain	9 (8.4%)
	Haematuria	18 (16.8%)
	Cloudy urine	8 (7.5%)
	Nocturia	4 (3.7%)
	Fever	4 (3.7%)
**No. of symptoms**	1	22 (20.6%)
	2	69 (64.5%)
	≥3	16 (15%)

^a^Data were presented as mean±SD for continuous variables and n (%) for categorical variables.

### Antibiotic resistance rates of *E*. *coli* isolates

Of the 107 *E*. *coli* isolates, the resistance rates were 59.8% for ampicillin, 31.8% for co-trimoxazole, 25.2% for gentamicin and 23.4% for ciprofloxacin. The majority of *E*. *coli* isolates were sensitive to nitrofurantoin (98.1%), amoxicillin (78.5%) and ciprofloxacin (76.6%) (Table 3).

**Table 3:** Susceptibility profile of *E*. *coli*, other uropathogens, ESBL producing isolates.

Antibiotic agents	*E*. *coli* isolates n = 107/141 (75.9%)			Other uropathogens isolates n = 34/141 (24.1%)			ESBL producing isolates n = 14/141 (9.9%)		
Susceptibility^a^ n (%)	S	I	R	S	I	R	S	I	R
Amoxicillin	84 (78.5%)	21 (19.6%)	2 (1.9%)	32 (94.1%)	1 (2.9%)	1 (2.9%)	6 (42.9%)	8 (57.1%)	0 (0%)
Ampicillin	41 (38.3%)	2 (1.9%)	64 (59.8%)	16 (47.1%)	0 (0%)	18 (52.9%)	0 (0%)	0 (0%)	14 (100%)
Ciprofloxacin	82 (76.6%)	0 (0%)	25 (23.4%)	31 (91.2%)	3 (8.8%)	0 (0%)	5 (35.7%)	1 (7.1%)	8 (57.1%)
Co-trimoxazole	73 (68.2%)	0 (0%)	34 (31.8%)	30 (88.2%)	0 (0%)	4 (11.8%)	5 (35.7%)	0 (0%)	9 (64.3%)
Gentamicin	80 (74.8%)	0 (0%)	27 (25.2%)	30 (88.2)	2 (5.9%)	2 (5.9%)	8 (57.1%)	0 (0%)	6 (42.9%)
Nitrofurantoin	105 (98.1%)	1 (0.9%)	1 (0.9%)	25 (73.5%)	6 (17.6%)	3 (8.8%)	12 (85.7%)	0 (0%)	2 (14.3%)

^a^S = sensitive; I = intermediate, R = resistant.

*E*. *coli* isolates with resistance to one drug can also be resistance to other drugs. Multidrug resistance (co-resistance to ampicillin, ciprofloxacin, and co-trimoxazole) occurred in 12.1% of *E*. *coli* isolates (n = 13).

### ESBL producers

ESBL producers (n = 14) were found in 9.9% of the positive isolates (n = 141) and almost all ESBL producers (n = 13) were from *E*. *coli* isolates. 85.7% (n = 12) of ESBL producing isolates were found amongst older women who were aged 51 years and above. All ESBL producing isolates were resistant to ampicillin, whilst almost half were resistant to ciprofloxacin (n = 8, 53.3%) and co-trimoxazole (n = 8, 53.3%).

### Antibiotic resistance rate of *E*. *coli* isolates and age and chronic medical conditions

Antibiotic resistance rates of ampicillin and multidrug resistance were statistically significant with increasing age. The OR of patients who aged >50 were 2.90 (95% CI: 1.28–6.55, *P* = 0.011) for having resistance with ampicillin. In addition, The OR of patients who aged >50 were 8.14 (95% CI: 1.02–65.26, *P* = 0.048) for having multidrug resistance. Meanwhile, although antibiotic resistance appear higher in those with more than two chronic medical conditions for most antibiotic types, this was not statistically significant (*P* = 0.134, *P* = 0.150*P* = 0.340 and *P* = 0.253 for ampicillin, ciprofloxacin, co-trimoxazole and multidrug resistance respectively) (Table 4).

**Table 4:** Odds ratio of patients’ characteristics and resistance to antibiotic agents (n = 107).

		Antibiotic resistance (*E*. *coli* isolates n = 107)			
		Ampicillin	Ciprofloxacin	Co-trimoxazole	Multi-drug resistance^a^
Patients’ characteristics		OR (95% CI) *P* value	OR (95% CI) *P* value	OR (95% CI) *P* value	OR (95% CI) *P* value
**Age group**	17–50	1.00	1.00	1.00	1.00
	>50	2.90 (1.28–6.55) 0.011^b^	2.13 (0.77–5.91) 0.145	2.40 (0.96–6.01) 0.062	8.14 (1.02–65.26) 0.048^b^
**No. of chronic medical conditions**	0	1.00	1.00	1.00	1.00
	1	2.68 (1.00–7.17) 0.050^b^	2.02 (0.76–5.39) 0.158	1.77 (0.70–4.42) 0.226	2.48 (0.72–8.49) 0.148
	≥2	2.75 (0.73–10.33) 0.134	3.42 (0.64–18.25) 0.150	1.94 (0.50–7.58) 0.340	3.65 (0.40–33.59) 0.253
**Past UTI diagnosis**	No	1.00	1.00	1.00	1.00
	Yes	1.19 (0.43–3.31) 0.743	3.04 (1.06–8.71) 0.033^b^	1.32 (0.47–3.72) 0.601	1.46 (0.36–5.92) 0.592
**Past antibiotic use**^**c**^	None	1.00	1.00	1.00	1.00
	In past 6 month	1.64 (0.53–5.11) 0.391	2.21 (0.71–6.86) 0.163	3.29 (1.11–9.80) 0.027^b^	2.96 (0.79–11.15) 0.096
	In past 1 year	NA	2.89 (1.09–7.68) 0.029^b^	2.43 (0.97–6.14) 0.056	3.29 (0.99–10.95) 0.043^b^

^a^Co-resistance to ampicillin, ciprofloxacin and co-trimoxazole.

^**b**^Statistically significant at *P*<0.05.

^**c**^Contains missing data.

### Factors associated with antibiotic resistance of *E*. *coli* isolates

Patients who had taken antibiotics in the past year had OR of 2.89 (95% CI: 1.09 to 7.68; *P* = 0.029) for resistance to ciprofloxacin than those without antibiotics treatment. In addition, the OR of those exposed to antibiotics in the past year were 3.29 (95% CI: 0.99 to 10.95; *P* = 0.043) to have multidrug resistance than those without previous antibiotics treatment (Table 4).

The OR for those with previous UTI diagnosis in the past 6 months were 3.04 (95%CI: 1.06 to 8.71, *P* = 0.03) to have ciprofloxacin resistance (Table 4).

### Antibiotic prescription

**Fig 2:**
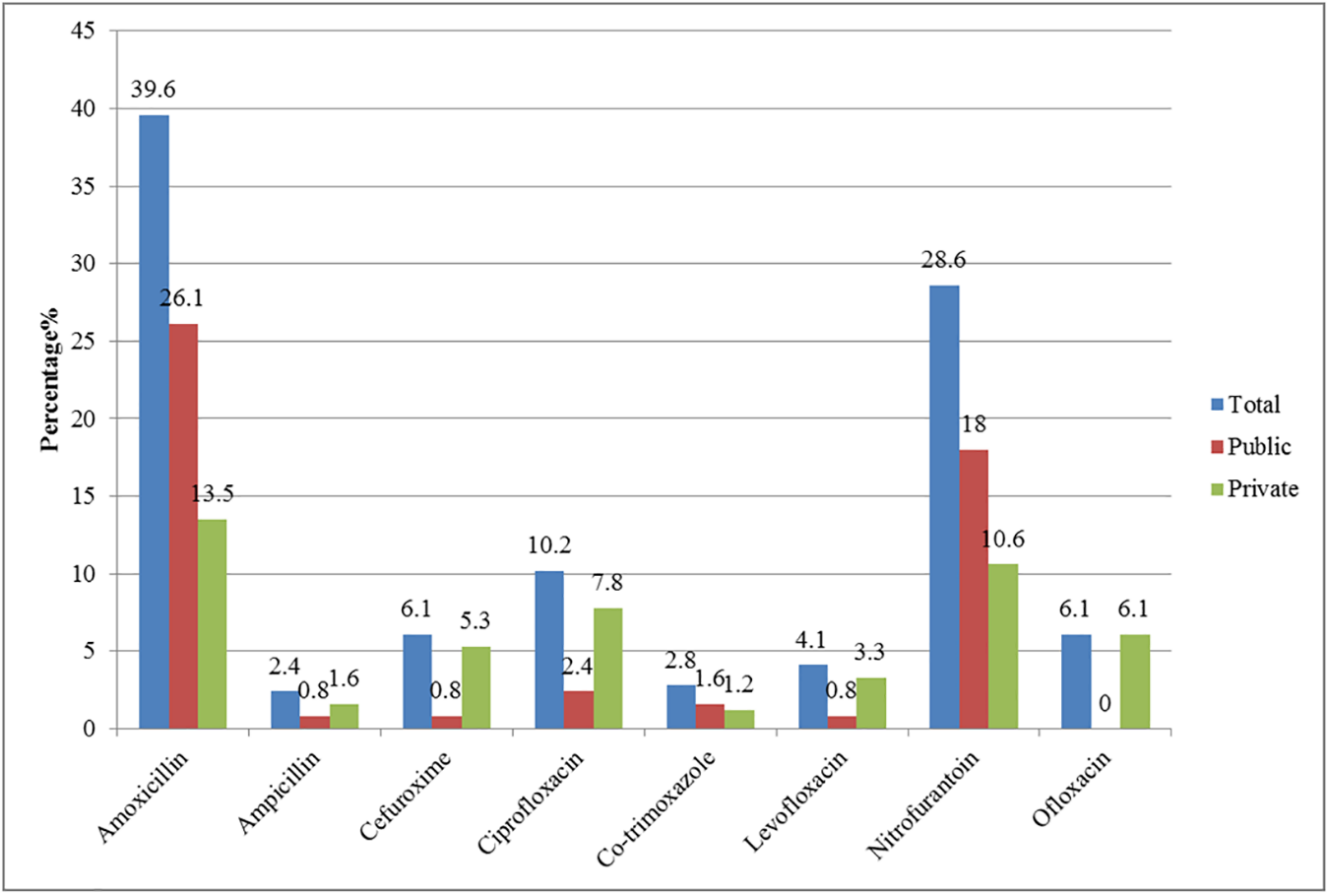
Antibiotic prescription rate among patients (n = 245).

Of the 298 patients presenting with symptoms, 245 (82.2%) were prescribed antibiotics. Amoxicillin (39.6%) was most commonly prescribed, followed by nitrofurantoin (28.6%) and ciprofloxacin (10.2%). The mean duration of antibiotic prescription was 6.86±0.54 days (Fig 2).

### Antibiotic prescription rates among public and private clinics

The antibiotic prescription rates were 50.6% and 49.4% in public and private primary care clinics respectively. There was no significant difference in empirical antibiotic prescription between the two groups (*P* = 0.147). Meanwhile, the OR of public primary care physicians to private primary care physicians in prescribing amoxicillin were 2.84 (95% CI: 1.67 to 4.85) and 2.01 (95% CI: 1.14 to 3.55) for nitrofurantoin. The results were statistically significant (*P*<0.001 and 0.015) (S1 Table).

In contrast, private primary care physicians prescribed more cefuroxime (5.3 versus 0.8%, *P* = 0.003) and ciprofloxacin (7.8 versus 2.4%, *P* = 0.005) (Fig 2).

### Factors for antibiotic prescription

There was no association between the rate of antibiotic prescription with age (*P* = 0.370). However, patients aged 65 years or above received more amoxicillin as empirical antibiotic treatment (*P* = 0.047), whilst patients aged between 17 and 50 years old received more cefuroxime (*P*<0.001) and ofloxacin (*P* = 0.005).

The OR of patients with co-existing medical conditions was 3.08 (95% CI: 1.60 to 5.94) to be prescribed amoxicillin than those without chronic medical conditions and this was significantly significant (*P* = 0.001).

The OR of prescribing ciprofloxacin was 2.17 (95% CI: 0.80 to 5.89, *P* = 0.122) and co-trimoxazole was 2.58 (95% CI: 0.48 to 13.84, *P* = 0.254) for patients with previous history of a UTI in the past 6 months than patients without a recent UTI. However the results were not statistically significant.

The odds of receiving an antibiotic prescription were greater with symptoms of dysuria or urgency. The OR of dysuria was 4.53 (95% CI: 2.33 to 8.83, *P*<0.001) and urgency (OR: 2.32, 95% CI: 1.18 to 4.58 *P* = 0.013). Meanwhile, The OR of patients presenting with more than two urinary symptoms were 0.33 (95% CI: 0.16 to 0.69, P = 0.003) time than those with one or two presenting symptoms to have empirical antibiotic treatment (S2 Table).

### Empirical antibiotic prescription and uropathogen susceptibility

For the 141 positive isolates, 122 received empirical antibiotic treatment. The appropriate use of antibiotics was evident in public primary care. This was demonstrated by the matching of antibiotic susceptibility of the uropathogen and the prescribed antibiotics were statistically significant for both sensitivity and resistance of *E*. *coli* pathogens and for other uropathogens. The OR in matching antibiotic susceptibility and physician prescribing was 6.72 (95% CI: 2.07 to 21.80, *P* = 0.001) for *E*. *coli* isolates and 6.19 (95% CI: 1.04 to 36.78, *P* = 0.034) for other uropathogens between public primary care and private primary care physicians (Table 5).

**Table 5:** Antibiotic prescription and uropathogen sensitivity and resistance.

	*E*.*coli* isolates n = 107		Other uropathogens n = 34	
	Public n = 47	Private n = 60	Public n = 13	Private n = 21
**Empirical antibiotics** **n (%)**	43 (91.5%)	49 (81.7%)	13 (100%)	17 (81%)
**No antibiotic prescribed** **n (%)**	4 (8.5%)	11 (18.3%)	0 (0%)	4 (19%)
**Antibiotic matching**^**a**^ **(overall)** **n (%)**	39/43 (90.7%)	29/49 (59.2%)	11/13 (84.6%)	8/17 (47.1%)
**OR (95% CI), *P* value**	6.72 (2.07–21.80), p = 0.001^c^	1.00	6.19 (1.04–36.78), p = 0.034^c^	1.00
**Antibiotic resistance**^**b**^ **(overall)** **n (%)**	1/43 (2.3%)	1/49 (2.0%)	0 (0%)	2/17 (11.8%)
**OR (95% CI), *P* value**	1.14 (0.07–18.84), p = 0.926	1.00	NA, p = 0.201	1.00

^a^Isolates were sensitive to physicians prescribed antibiotics (amoxicillin, ampicillin, ciprofloxacin, co-trimoxazole, gentamicin and nitrofurantoin).

^b^Isolates were resistant to physicians prescribed antibiotics (amoxicillin, ampicillin, ciprofloxacin, co-trimoxazole, gentamicin and nitrofurantoin).

^c^Statistically significant at *P*<0.05.

## Discussion

### Antibiotic resistance

Our result showed that *E*. *coli* was the most common pathogen and present in 75.9% of the positive urine cultures, which was consistent with international studies [3,4,12–14].

Regarding antibiotic sensitivity and resistance rate, our results were consistent with a regional study of urinary isolates of outpatients from private hospital laboratories and community laboratories, the sensitivity rates of ampicillin (38.3% versus 37.4–37.6%), ciprofloxacin (76.6% versus 76.3–77.2%), co-trimoxamole (68.2% versus 64.7–65%) were similar. In addition, a lower antibiotic sensitivity to amoxicillin (Sensitivity rates of 81.3%-83.6% vs 78.5%) was observed, although resistance rates were low and comparable [10].

Reduced susceptibility to amoxicillin in our study may indicate patients presenting to primary care which may have less severe symptoms and likely to present earlier or may reflect changes in antibiotic susceptibilities due to physicians’ prescribing behaviour. The Centre of Health Protection in Hong Kong publishes sentinel surveillance information of outpatient urinary samples every month [15]. Meanwhile the sentinel data analyses around 500 routine samples per month, all of which are urine samples collected in 85 public primary care centres (GOPCs); this includes male and female who are likely to those who have complicated or recurrent urinary illness to warrant further investigation from urinary microscopy and culture. Over comparable periods in 2012–13, bacterial pathogens and antibiotic resistance to most antibiotics were similar to our results. However, the antibiotic resistance rates for co-trimoxazole as well as ESBL producers were lower in our study (31.8 versus 38% and 9.9% versus 18.0% respectively). Meanwhile, the website publishes resistance rates only, whilst sensitive and intermediate responses may also be helpful in primary care physicians choice of antibiotics given other factors e.g. co-morbidities and contraindication.

Previous studies have suggested an association between antibiotic resistance and increasing age [10] and whilst this trend was observed, only a significant finding of age and resistance to ampicillin and multiple drugs were evident in our study. Our study showed that public primary care clinics and past history of UTI diagnosis (within 6 months) were statistically significant associated with *E*. *coli* isolates resistance to ciprofloxacin. A study in Europe suggests that the prior use of antibiotics may be a contributory factor for antibiotic resistance; Sotto et al [16] found that a past diagnosis of UTI within the past 1 year was associated with *E*. *coli* antibiotic resistance to ciprofloxacin.

### Antibiotic prescription

The results of our study suggest that nitrofurantoin is the most appropriate first line antibiotic of choice which had the lowest rate of resistance and the highest sensitivity. Second line treatment would be amoxicillin. Our result is similar with current guidelines on antibiotic prescription for UTI, such as Infectious Diseases Society of America guidelines [17,18] and inter-hospital guidelines in Hong Kong [19]. High antibiotic resistance rates to ampicillin, ciprofloxacin and co-trimoxazole (resistance rate > 20%) have been consistent to a local study [20] and demonstrate that these drugs should not be considered in the management of UTI unless there is proven resistance or allergy to nitrofurantoin or amoxicillin.

In our study, the most commonly prescribed antibiotic amongst primary care physicians is amoxicillin followed by nitrofurantoin. Selection of appropriate antibiotics should be based on differing resistance patterns in the locality [21], as well as patients’ medication, contraindications, allergy profile and cost [18]. All these considerations may affect the antibiotic prescription pattern, which may account for the differences in the antibiotic prescription rate. The use of nitrofurantoin may be limited as it is contraindicated in patients with renal impairment and creatinine clearance <60mL/min and may require careful consideration in the elderly [22] and this may explain the reluctance in prescribing. The threshold of creatinine clearance <60mL/min however, has been disputed due to non-existent evidence and suggests a lower threshold of creatinine clearance <40mL/min could be adapted [23] to improve nitrofurantoin use. Meanwhile, even with cautionary use, it may be likely that nitrofurantoin is underutilised and further exploration in clinician’s reluctance and other factors involved in the antibiotic decision making process.

Meanwhile, our result shows that private primary care physicians prescribed more ciprofloxacin (*P* = 0.005), while public primary care physicians prescribed more amoxicillin (*P*<0.001) and nitrofurantoin (*P* = 0.015). This may be attributed to guidelines, drug formulary and the cost of treatment. The public primary care clinics are governed by the Hospital Authority which has guidance for public primary care physicians on their clinical management system. Private group practices have their own clinical management systems and are not restricted by guidelines and have a wider drug formulary. Meanwhile, private group practices are able to absorb higher drug costs. In Hong Kong, a 3-day course of nitrofurantoin and ciprofloxacin costs 2.38 and 25.8 HKD respectively (1 USD = 7.8 HKD) [24]. The predominant use of nitrofurantoin and amoxicillin in public clinics may also be attributed to the use of available inter-hospital guidance [19] and clinical governance of its governing body the Hospital Authority and may reflect the benefits of governance on antibiotic prescribing behaviour. Surveillance of prescribing data of private doctors may enhance appropriate antibiotic prescribing, but this would logistically be difficult due to the heterogeneity of clinical systems and documentation by private practitioners and lack of a prescription tracking system as many private practitioners are dispensing practices.

International recommendations for treating uncomplicated UTI in primary care is a 3 day course of antibiotics [25], however 99.5% of the antibiotic prescriptions in this study were of 5–7 days duration. Our results show that patients who attended private clinics were younger (mean 49.50±17.66 years old) and had less comorbidity when compared to patients who attended public clinics (Table 1). Private primary care physicians also prescribed cefuroxime and ofloxacin which has been documented for use in young women [26,27]. Further exploration of physician’s prescribing behaviour and facilitators and barriers to appropriate prescribing is warranted.

### Strength and limitations

This is the first study to investigate both antibiotic resistance and prescription rates of patients with uncomplicated UTI in Chinese primary care settings. Previous antibiotic resistance data was conducted in outpatients departments and public primary care clinics. Meanwhile, there have been no studies documenting physician prescribing habits in the region. The strength of this study has been to show the use of both antibiotic susceptibility data and antibiotic prescribing behaviour in identifying highlighting gaps associated with antibiotic appropriateness.

This study has several limitations. The participation of group practices in selected districts may limit the representativeness of patients across the Hong Kong S.A.R region. Due to the uncommon and sporadic presentation for UTI across a number of sites, the recruitment period was long and we were unable to document the number of patients who were approached but declined to participate in the study which may attribute to selection bias of the study sample. In addition, primary care physicians may also be more likely to prescribe an appropriate antibiotic as they were aware of being observed. Recall bias may also occur in patients recounting prior UTI episodes and prior antibiotic use. However these limitations are likely to be underestimating our study findings. The study was designed to reflect the pragmatic approach of UTI diagnosis and empirical antibiotic prescription in primary care. The use of urine dipstick in the diagnostic criteria, the follow up of presenting symptoms and analysis of urine culture yields of 10^3^ and 10^4^ cfu/ml may further enhance the study, particularly in detailing threshold for treatment and symptom resolution.

Our study has highlighted a gap between physician current prescribing habits and antibiotic resistance. There is a need for increased efforts for education and dissemination to improve appropriate antibiotic prescription. It may also be advantageous for the surveillance data to show intermediate susceptibility to particular antibiotics as well as resistance data to help guide primary care physicians on antibiotic choice. The prospective cohort and multicentre approach in this study yields a more comprehensive picture of the complexities in the patient presentation and doctors’ prescription of antibiotics not addressed in previous regional studies. Additional information on patient’s clinical response and recovery and physician’s decision on antibiotic prescription and choice can be considered in further studies.

## Conclusions

This study identified antibiotic resistance amongst uropathogens and physician antibiotic prescription in primary care settings. There is scope to encourage nitrofurantoin as first line treatment in primary care settings particularly in private primary care and to explore its use and barriers for use. Enhancing antibiotic guidance for UTI and continued surveillance of antibiotic resistance and physician antibiotic prescription may improve appropriate antibiotic prescription for UTI in Chinese primary care.

## Supporting information

S1 Table Odds ratio for clinics and antibiotic prescription. ^a^Statistically significant at P<0.05.
(PDF)

S2 Table Odds ratio for presenting symptoms for UTI and antibiotic prescription. ^a^Statistically significant at P<0.05.
(PDF)
