# Correction: Antibiotic resistance rates and physician antibiotic prescription patterns of uncomplicated urinary tract infections in southern Chinese primary care

**DOI:** 10.1371/journal.pone.0192466

**Published:** 2018-02-23

**Authors:** Carmen Ka Man Wong, Kenny Kung, Philip Lung Wai Au-Doung, Margaret Ip, Nelson Lee, Alice Fung, Samuel Yeung Shan Wong

The antibiotic name amoxicillin appears incorrectly throughout the article. The correct antibiotic name is amoxicillin-clavulanate.

The antibiotic name amoxicillin appears incorrectly in Tables 3 and 5. Please see the correct Tables 3 and 5 below.

**Table 3:** Susceptibility profile of E. coli, other uropathogens, ESBL producing isolates.

Antibiotic agents	*E*. *coli* isolates n = 107/141 (75.9%)			Other uropathogens isolates n = 34/141 (24.1%)			ESBL producing isolates n = 14/141 (9.9%)		
Susceptibility^a^ n (%)	S	I	R	S	I	R	S	I	R
Amoxicillin-clavulanate	84 (78.5%)	21 (19.6%)	2 (1.9%)	32 (94.1%)	1 (2.9%)	1 (2.9%)	6 (42.9%)	8 (57.1%)	0 (0%)
Ampicillin	41 (38.3%)	2 (1.9%)	64 (59.8%)	16 (47.1%)	0 (0%)	18 (52.9%)	0 (0%)	0 (0%)	14 (100%)
Ciprofloxacin	82 (76.6%)	0 (0%)	25 (23.4%)	31 (91.2%)	3 (8.8%)	0 (0%)	5 (35.7%)	1 (7.1%)	8 (57.1%)
Co-trimoxazole	73 (68.2%)	0 (0%)	34 (31.8%)	30 (88.2%)	0 (0%)	4 (11.8%)	5 (35.7%)	0 (0%)	9 (64.3%)
Gentamicin	80 (74.8%)	0 (0%)	27 (25.2%)	30 (88.2)	2 (5.9%)	2 (5.9%)	8 (57.1%)	0 (0%)	6 (42.9%)
Nitrofurantoin	105 (98.1%)	1 (0.9%)	1 (0.9%)	25 (73.5%)	6 (17.6%)	3 (8.8%)	12 (85.7%)	0 (0%)	2 (14.3%)

^a^S = sensitive; I = intermediate, R = resistant.

**Table 5:** Antibiotic prescription and uropathogen sensitivity and resistance.

	*E*.*coli* isolates n = 107		Other uropathogens n = 34	
	Public n = 47	Private n = 60	Public n = 13	Private n = 21
**Empirical antibiotics** **n (%)**	43 (91.5%)	49 (81.7%)	13 (100%)	17 (81%)
**No antibiotic prescribed** **n (%)**	4 (8.5%)	11 (18.3%)	0 (0%)	4 (19%)
**Antibiotic matching**^a^ **(overall)** **n (%)**	39/43 (90.7%)	29/49 (59.2%)	11/13 (84.6%)	8/17 (47.1%)
**OR (95% CI), *P* value**	6.72 (2.07–21.80), p = 0.001^c^	1.00	6.19 (1.04–36.78), p = 0.034^c^	1.00
**Antibiotic resistance**^b^ **(overall)** **n (%)**	1/43 (2.3%)	1/49 (2.0%)	0 (0%)	2/17 (11.8%)
**OR (95% CI), *P* value**	1.14 (0.07–18.84), p = 0.926	1.00	NA, p = 0.201	1.00

^a^Isolates were sensitive to physicians prescribed antibiotics (amoxicillin-clavulanate, ampicillin, ciprofloxacin, co-trimoxazole, gentamicin and nitrofurantoin).

^b^Isolates were resistant to physicians prescribed antibiotics (amoxicillin-clavulanate, ampicillin, ciprofloxacin, co-trimoxazole, gentamicin and nitrofurantoin).

^c^Statistically significant at *P*<0.05.

The antibiotic name amoxicillin appears incorrectly in Fig 2. The authors have provided the corrected version here.

**Fig 2:**
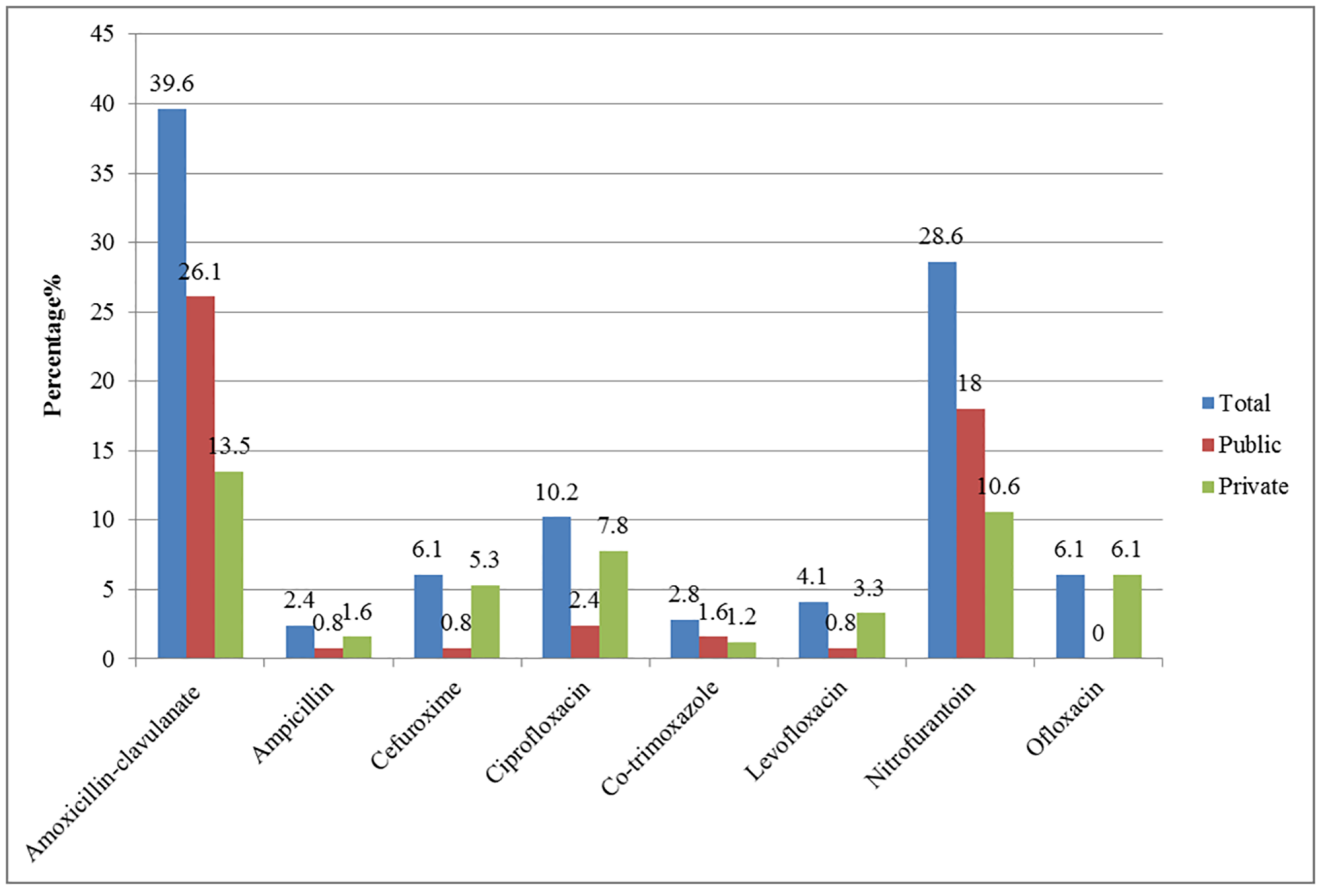
Antibiotic prescription rate among patients (n = 245).

## References

[1] Wong CKM, Kung K, Au-Doung PLW, Ip M, Lee N, Fung A, et al (2017) Antibiotic resistance rates and physician antibiotic prescription patterns of uncomplicated urinary tract infections in southern Chinese primary care. PLoS ONE 12(5): e0177266 https://doi.org/10.1371/journal.pone.0177266 28486532 10.1371/journal.pone.0177266 PMC5423680

