# Supplementary material for: Antibiotic resistance rates and physician antibiotic prescription patterns of uncomplicated urinary tract infections in southern Chinese primary care
Source: PLoS One. 2017 May 9;12(5):e0177266. doi: 10.1371/journal.pone.0177266 (PMC5423680; doi:10.1371/journal.pone.0177266)
Supplement: S1 Table — aStatistically significant at P<0.05. (PDF) [file pone.0177266.s001.pdf]

S1 Table. Odds ratio for clinics and antibiotic prescription (n=245).

Antibiotic agents	Clinics	OR (95% CI)	*P* value
**Empirical antibiotic treatment**	Private	1.00	
	Public	1.56 (0.85-2.86)	0.147
**Amoxicillin**	Private	1.00	
	Public	2.84 (1.67-4.85)	<0.001[a]
**Ampicillin**	Private	1.00	
	Public	0.48 (0.09-2.67)	0.391
**Cefuroxime**	Private	1.00	
	Public	0.14 (0.03-0.62)	0.003[a]
**Ciprofloxacin**	Private	1.00	
	Public	0.27 (0.11-0.71)	0.005[a]
**Co-trimoxazole**	Private	1.00	
	Public	1.31 (0.29-5.99)	0.726
**Levofloxacin**	Private	1.00	
	Public	0.23 (0.05-1.11)	0.048[a]
**Nitrofurantoin**	Private	1.00	
	Public	2.01 (1.14-3.55)	0.015[a]
**Ofloxacin**	Private	NA	<0.001[a]
	Public		

[a]Statistically significant at $P<0.05$.
