# Supplementary material for: Antibiotic resistance rates and physician antibiotic prescription patterns of uncomplicated urinary tract infections in southern Chinese primary care
Source: PLoS One. 2017 May 9;12(5):e0177266. doi: 10.1371/journal.pone.0177266 (PMC5423680; doi:10.1371/journal.pone.0177266)
Supplement: S2 Table — aStatistically significant at P<0.05. (PDF) [file pone.0177266.s002.pdf]

S2 Table. Odds ratio for presenting symptoms for UTI and antibiotic prescription (n=298).

Symptoms	Presence of symptoms	OR (95% CI)	*P* value
**Dysuria**	Absent	1.00	
	Present	4.53 (2.33-8.83)	<0.001[a]
**Fever**	Absent	1.00	
	Present	0.81 (0.09-7.38)	0.849
**Frequency**	Absent	1.00	
	Present	1.74 (0.95-3.21)	0.073
**Cloudy urine**	Absent	1.00	
	Present	1.09 (0.30-3.88)	0.899
**Nocturia**	Absent	1.00	
	Present	0.51 (0.13-2.01)	0.329
**Abdominal/loin pain**	Absent	1.00	
	Present	1.20 (0.51-2.87)	0.674
**Urgency**	Absent	1.00	
	Present	2.32 (1.18-4.58)	0.013[a]
**Haematuria**	Absent	1.00	
	Present	0.96 (0.42-2.21)	0.931
**No of the above symptoms**	1-2 symptoms	1.00	
	>2 symptoms	0.33 (0.16-0.69)	0.003[a]

[a]Statistically significant at $P<0.05$.
